# The Association between Early Artificial Amniotomy and Chorioamnionitis in Nulliparous Induction of Labor

**DOI:** 10.1155/2014/628452

**Published:** 2014-12-16

**Authors:** Laura G. Cooney, Jamie A. Bastek

**Affiliations:** ^1^Maternal and Child Health Research Program, Department of Obstetrics and Gynecology, Center for Research on Reproduction and Women's Health Perelman School of Medicine, University of Pennsylvania, Philadelphia, PA 19104, USA; ^2^Division of Maternal-Fetal Medicine, Department of Obstetrics and Gynecology, Hospital of the University of Pennsylvania, 3400 Spruce Street, 585 Dulles Building, Philadelphia, PA 19104, USA

## Abstract

*Objectives*. To investigate whether early artificial amniotomy (AROM) <4 cm in nulliparous women admitted for induction of labor was associated with an increased rate of chorioamnionitis and cesarean section or a decreased time to vaginal delivery. *Study Design*. A retrospective cohort study was performed on nulliparous women with a term, singleton gestation and intact membranes who presented for induction of labor (January 2008 to December 2011). Chorioamnionitis was defined using ICD9 codes. *Results*. 1,567 women were enrolled; 25.4% underwent early AROM. Overall, the prevalence of chorioamnionitis was 12.4%, the rate of cesarean section was 32.2%, and the time from 4 cm cervical dilation to vaginal delivery was 413 min. Compared to women without AROM < 4 cm, early AROM did not affect overall chorioamnionitis rates (10.2 versus 13.2%, *P* = 0.12) but was associated with an increased cesarean section rate (40.2 versus 29.5%, *P* < 0.001). However, among those who delivered vaginally, AROM < 4 cm decreased the rate of chorioamnionitis (8.4 versus 14.6%, *P* = 0.01), which persisted when controlling for potential confounders (OR 0.55, 95% CI 0.33–0.92), and decreased the time from 4 cm dilation to vaginal delivery (329 versus 472 min, *P* < 0.001). *Conclusions*. Our findings do not suggest that early AROM is associated with an increased rate of clinical chorioamnionitis.

## 1. Introduction

Induction of labor is becoming more common with over 23% of pregnant women being induced in 2010 [1]. Women who undergo an induction of labor have increased rates of maternal and neonatal morbidity [2] making research on safe and efficacious induction agents a priority.

Artificial rupture of membranes (amniotomy) is included in the active management of labor in multiparous women [3] and may shorten the duration of labor in nulliparous women who present in spontaneous labor [4–7]. Similarly, a few studies evaluating the use of early artificial amniotomy as an induction agent in nulliparous women have demonstrated potential benefits such as a decreased time to delivery. However, the reports on the effect of early amniotomy on complications such as chorioamnionitis vary [8–11].

Research on interventions that impact rates of chorioamnionitis is important as chorioamnionitis has been associated with significant maternal and neonatal morbidity including postpartum hemorrhage, maternal and neonatal sepsis, longer hospital stays, and the infant's future development of cerebral palsy [12, 13]. While there is a known increase in the rate of chorioamnionitis in patients exposed to premature spontaneous rupture of membranes [14, 15], none of the randomized controlled trials on early artificial rupture of membranes have been powered to detect differences between the rates of chorioamnionitis in the early and late amniotomy groups as their primary outcome [8–11]. Therefore, our primary objective was to assess whether exposure to early artificial amniotomy, defined as a cervical dilatation < 4 cm, was associated with an increased rate of chorioamnionitis in nulliparous women undergoing an induction of labor at term as compared to women without this exposure.

Prior studies have noted a decreased time to delivery of between two and four hours with early amniotomy but have shown conflicting results on the rate of cesarean section [8–11]. Therefore, our secondary objective was to compare the length of time to vaginal delivery and the rate of cesarean section between the two groups.

We hypothesized that patients exposed to early artificial amniotomy would have an increased rate of chorioamnionitis, a decreased time to vaginal delivery, and an increased risk of cesarean section.

## 2. Materials and Methods

We performed a retrospective cohort study at a single, urban, tertiary care center including patients who presented for delivery between January 2008 and December 2011. The cohort consisted of pregnant women who had not had a prior delivery and who were admitted for induction of labor with intact membranes and a term (gestational age ≥ 37 0/7 weeks), singleton fetus. We excluded women who presented with a cervical dilation ≥ 4 cm, who had spontaneous rupture of membranes at a cervical dilation < 4 cm, who were diagnosed with chorioamnionitis prior to admission, or who had a delivery by cesarean section without a trial of labor. To study our prespecified secondary outcome of time to vaginal delivery, we excluded patients who delivered by cesarean section.

Of note, women with spontaneous rupture of membranes at <4 cm were excluded from our study because they did not have the opportunity to be exposed to our intervention, early artificial amniotomy. However, women who presented with intact membranes and a cervical exam < 4 cm and subsequently underwent spontaneous rupture of membranes at ≥4 cm were included in the late amniotomy group along with women with artificial amniotomy at ≥4 cm.

Early artificial amniotomy was defined as a cervical dilation < 4 cm at the time of amniotomy. Timing of amniotomy at our institution is determined by the covering providers, a resident physician in conjunction with an attending physician. The attending physicians staffing our labor floor include both general obstetricians and maternal fetal medicine specialists. Four centimeters was chosen because it was previously thought to be the cervical exam differentiating latent and active labor based on the Friedman curve [16]. Although recent studies have demonstrated that active labor in nulliparous women might not occur until a more advanced cervical exam [17], 4 cm is likely the earliest that this transition would occur and therefore remains a cut-point at which an intervention such as amniotomy could positively or negatively impact the labor process.

At our institution, chorioamnionitis is strictly defined as a maternal temperature ≥ 38°C and at least one of the following: maternal tachycardia (≥100 beats per minute), fetal tachycardia (≥160 beats per minute), and/or fundal tenderness. The diagnosis of chorioamnionitis and the criteria by which this diagnosis is made are documented by the diagnosing physician in the medical chart.

All patients at our institution with chorioamnionitis are treated with ampicillin sulbactam or, in the setting of a penicillin allergy, with gentamicin and clindamycin. All women undergoing a cesarean section receive cefazolin (or gentamicin/clindamycin) prior to skin incision. Patients diagnosed with chorioamnionitis prior to receiving a cesarean section are continued on ampicillin sulbactam (or gentamicin/clindamycin) for 24 hours after the procedure. Endometritis is treated with ampicillin sulbactam (or gentamicin/clindamycin). Women in our practice are universally screened for group B streptococcus (GBS) infections during prenatal care and antibiotics for GBS prophylaxis during labor are administered according to ACOG guidelines [18]. Positive GBS status does not affect the timing of amniotomy at our institution.

Our primary outcome was the rate of chorioamnionitis as identified using ICD-9 codes. The data abstractors who identified the diagnosis of chorioamnionitis were trained professionals looking for physician documentation of clinical chorioamnionitis. Our secondary outcomes included the time to vaginal delivery, the rate of cesarean section, the rate of postpartum endometritis, neonatal 5-minute APGAR scores, and neonatal admission to the intensive care nursery. In the subpopulation of women who delivered vaginally, time to vaginal delivery was defined as time from the first cervical exam of 4 cm dilation to vaginal delivery. At our institution, endometritis is defined as the presence of both a maternal temperature ≥ 38°C at least 24 hours after delivery and fundal tenderness. Endometritis was identified using ICD-9 codes.

### 2.1. Data Collection

Relevant obstetric information for all patients who delivered at our institution between January 2008 and December 2011 was charted in an electronic obstetric system (Centricity Perinatal database) and delivery information during this time was entered by the delivering physician into a separate electronic database (BabyTracker). Demographic information, indication for induction of labor, and delivery details were extracted from these two databases. ICD-9 codes were used for the diagnosis of chorioamnionitis and endometritis and for the identification of relevant maternal medical conditions as this information is not routinely recorded in either the Centricity Perinatal or BabyTracker databases. The total number of cervical exams, cervical exams at specific time points, and the use of a fetal scalp electrode and/or an intrauterine pressure catheter were unable to be electronically extracted; thus they were obtained from a retrospective review of the Centricity Perinatal database. At our institution, the nurse records the cervical exam in Centricity Perinatal database at the bedside while the provider is performing the exam. When there was no cervical exam recorded at exactly 4 cm dilation, the approximate time when patient reached 4 cm dilation was extrapolated based on the cervical dilation at the preceding and subsequent cervical exams. Cervical consistency and position are not routinely recorded with cervical exams and thus no Bishop scores could be calculated.

### 2.2. Statistical Methods

We performed* a priori* sample size calculations, powered to our primary outcome of chorioamnionitis. Based on unpublished data from our institution, we assumed a prevalence of chorioamnionitis at term of approximately 5%, a Type I error rate of 5%, 80% power, and an enrollment ratio of 1 : 4 between those exposed versus those not exposed to early artificial amniotomy. Under these assumptions, we estimated that we would need approximately 1,570 patients to detect a 2-fold difference in the rate of chorioamnionitis between exposure groups.

We compared categorical data with chi-squared tests, continuous data with Student's *t*-tests, or nonparametric comparison of medians as appropriate and determined the odds of chorioamnionitis controlling for confounders with multivariable logistic regression. STATA version 10.1 (College Station, TX) was used to perform all analyses. A *P* value of <0.05 was considered significant for all analyses.

This study was approved by the Institutional Review Board at the University of Pennsylvania.

## 3. Results

A total of 16,608 women delivered at our university between January 2008 and December 2011, of whom 1,567 met inclusion criteria and were included in our analyses. 398 women (25.4%) were classified as exposed to early artificial amniotomy, while 1,169 women (74.6%) were classified as unexposed. The median cervical dilation at amniotomy was 3 cm (IQR 3.0–3.5) in the early amniotomy group compared to 4.5 cm (IQR 4.0–5.2) in the group without early amniotomy. All of the women in the early amniotomy group had an artificial amniotomy, while 80.4% of women in the alternate group had an artificial amniotomy and 19.6% had a spontaneous amniotomy at ≥4 cm dilation.

The groups were well balanced with regard to demographics and preexisting medical conditions (Table 1). Both groups had similar indications for induction of labor including maternal (hypertensive disorders, diabetes, advanced maternal age > 40 years, and elective or another maternal medical condition), fetal (intrauterine growth restriction, oligohydramnios, polyhydramnios, or nonreassuring fetal status), and concurrent maternal and fetal indications for induction. Rates of maternal GBS positive status were similar in each group. The rates of each induction method and labor intervention were compared and were remarkable for higher rates of need for cervical ripening, specifically prostaglandin use, and placement of a fetal scalp electrode and/or an intrauterine pressure catheter in the early amniotomy group. The proportion of women receiving early amniotomy varied by year: early amniotomy was more prevalent in 2008 and 2011 compared to 2009 and 2010.

Overall, the rate of chorioamnionitis was 12.4% and did not change with exposure to early artificial amniotomy (10.2 versus 13.2%; *P* = 0.12) (Table 2). However, within the cohort of women who delivered vaginally, there was a statistically significant 57.5% decrease (8.4 versus 14.6%, *P* = 0.01) in the rate of chorioamnionitis in the early amniotomy group. This decrease did not translate into an increase in the rate of chorioamnionitis among those in the early amniotomy group who underwent a cesarean section (12.7 versus 9.7%, *P* = 0.3).

Associations between demographic variables of interest and chorioamnionitis are included in Table 3. Location of prenatal care and the use of epidural anesthesia were associated with increased rates of chorioamnionitis, while use of intrauterine pressure catheters, fetal scalp electrodes, maternal GBS positive status, and need for cervical ripening were not. Although no variables were associated with both early amniotomy and chorioamnionitis, we identified variables that were associated with either early amniotomy or chorioamnionitis as potential confounders. Controlling for these potential confounders (intrauterine pressure catheter, fetal scalp electrode, induction agent, year of delivery, and epidural use), the association between early artificial amniotomy and chorioamnionitis persisted in the subset who delivered vaginally (OR 0.56, 95% CI 0.34–0.93) (Table 5). This association between early artificial amniotomy and chorioamnionitis was not significant in the overall population when controlling for potential confounders associated with chorioamnionitis (*P* < 0.1) including location of prenatal care, gestational age, use of epidural, meconium stained amniotic fluid, and cervical ripening (OR 0.76, 95% CI 0.52–1.10) (Table 5).

Associations between early amniotomy and relevant secondary outcomes are listed in Table 2. 1,062 women (67.8%) had a vaginal delivery. Among those with a vaginal delivery, the overall time from 4 cm cervical dilation to delivery was 413 minutes. Early amniotomy at <4 cm dilation was associated with an approximately 2 hours and 20 minutes decreased time from 4 cm dilation to delivery (*P* < 0.001).

The overall rate of cesarean section was 32.2%. The rate of cesarean section was higher among those exposed to early artificial amniotomy (40.2 versus 29.5%; *P* < 0.001). When comparing the indication for cesarean section (Table 4), early artificial amniotomy was associated with fewer cesarean sections performed for active phase labor dystocia (defined as arrest of active phase or arrest of descent) and more cesarean sections for nonreassuring fetal status or latent phase labor dystocia (defined as failed induction or failure to progress), but none of these differences were statistically significant. Among patients with a cesarean section, neonatal weight was 112 grams lower in the early compared to the late amniotomy group (*P* = 0.02). The overall rate of endometritis was 1.3%. The rate of endometritis was increased in the early amniotomy group (2.3 versus 0.9%, *P* = 0.04); however, the absolute risk remained low.

There were no significant differences in median 5-minute APGAR scores (9 versus 9, *P* = 0.13) or the rate of admission to the neonatal intensive care nursery (14.3 versus 14.2%, *P* = 0.95) between the exposed and unexposed groups (Table 2).

## 4. Discussion

Our study is the first to evaluate the rate of chorioamnionitis as the primary outcome in nulliparous women undergoing an induction of labor at term comparing those exposed versus those not exposed to early artificial amniotomy. We found that early artificial amniotomy did not increase the rate of chorioamnionitis. In addition, this intervention more than halved the rate of chorioamnionitis among those who delivered vaginally. Although maternal GBS positive status and IUPC use have been associated with increased rates of chorioamnionitis in other studies [19, 20] we did not find this association in our study.

We also demonstrated that early artificial amniotomy significantly decreased the time to vaginal delivery by more than 2 hours. Not only is this relevant for labor floor turnover, but also this may decrease the rate of unstudied maternal morbidities among those for whom expedited delivery is most critical.

Prior randomized controlled trials evaluating early artificial amniotomy and induction of labor have been powered to primary outcomes of either time to delivery [8, 11] or the rate of cesarean section [9, 10]. Of these, only two studies specifically looked at outcomes in nulliparous women [8, 9]. Neither of these studies showed a significant difference in the rate of chorioamnionitis between groups, although Gagnon-Gervais et al. showed a trend toward a decrease in the rate of intrapartum fever (6 versus 25%, *P* = 0.05) [9] while MacOnes et al. showed a nonsignificant increase in chorioamnionitis (11.5 versus 8.5%, *P* = 0.22) [8]. Although our 12.5% overall rate of chorioamnionitis is higher than the 5% average rate of chorioamnionitis for all patients who deliver at our institution (unpublished data), it is comparable to those rates of chorioamnionitis in studies with a similar population [8, 9]. Similarly, our rates of admission to the neonatal intensive care nursery (14.3 versus 14.2%) are also comparable to rates noted by MacOnes et al. (13.6 versus 15.0%) [8].

Previously published data is inconsistent regarding the association between early amniotomy and the risk of cesarean section [8–11]. While we found an overall increased rate of cesarean section, this could not be attributed to an increase in cesarean sections performed for either dystocia or nonreassuring fetal status. Although macrosomia can be associated with increased cesarean section rates, there was no difference in neonatal weights in the early versus late amniotomy groups. The 112-gram decrease in neonatal weight in the women in the early amniotomy group who underwent a cesarean section, although statistically significant, is not clinically relevant.

Our study has several strengths. First, based on a PUBMED search of the words “amniotomy” or “artificial rupture of membranes” along with either “fever,” “chorioamnionitis,” “nulliparous,” or “induction of labor,” which was performed in March 2012 and reaffirmed in March 2014, our study is the largest cohort of nulliparous women studied to date and the first to be powered to the rate of chorioamnionitis as the primary outcome. In addition, all patients were enrolled from a single institution thereby increasing the consistency of care between patients. Finally, our decrease in time to delivery is similar to that seen in other studies on early amniotomy in nulliparous induction of labor: 2.3 hours [8] and 4.1 hours [9]. Our rates of chorioamnionitis and neonatal intensive nursery admission are also similar to these studies [8, 9] supporting the reproducibility of our findings.

Our study was not without weaknesses. The retrospective nature makes it impossible to prove causation, only an association, between early artificial amniotomy and outcomes of interest. As patients were not randomized, the decision to perform amniotomy was based upon provider preference and may be performed not only as part of normal labor induction but also during the management of preexisting nonreassuring fetal status or labor dystocia. This provider bias may have influenced our results, particularly the increased rate of cesarean section. It is noteworthy that prior randomized controlled trials have not found an increase in cesarean section rates when amniotomy is used as an induction agent [8, 9, 11], except when performed immediately after induction with a foley balloon and in the absence of regular contractions [10]. Provider bias is also evident in the variation in the rate of early artificial amniotomy by year. Maternal-fetal medicine specialists covered the labor floor with greater frequency prior to 2009 and again in 2011; from 2009 to 2010 there was greater generalist and laborist coverage.

Our definitions of chorioamnionitis and endometritis were derived from ICD-9 codes rather than culture proven infections. However, given the uniformity of the definition employed by providers in making this diagnosis and the data abstraction by trained professionals, we believe our findings should still be generalizable.

We defined length of labor as time from a cervical dilation of 4 cm to delivery. The time from use of first induction agent to delivery might be a more uniform way of evaluating the effect of early amniotomy on the length of labor; however, it was difficult to determine the start time of induction in a retrospective fashion. Despite this limitation, we do not believe that the amount of time that each group labored before 4 cm dilation should differ significantly and thus impact our results.

Finally, as with other studies on early amniotomy, we lack good indicators of neonatal outcomes. We only analyzed short-term outcomes such as APGAR scores and nursery acuity, but rates of neonatal sepsis and poor neurodevelopmental outcomes would be better measures of the true risks or benefits of early amniotomy. Unfortunately, due to the fact that neonatal data is captured under the infant's name and medical record number, which cannot be linked to maternal data at our institution, abstracting this information from chart review was beyond the scope of this study.

Our findings suggest that early artificial amniotomy does not increase the rate of chorioamnionitis but does decrease the time to vaginal delivery. This should be considered when managing patients for whom a decreased length of labor may be critical.

## 5. Conclusions

In nulliparous women undergoing an induction of labor, early artificial amniotomy does not increase the rate of clinical chorioamnionitis.

## Figures and Tables

**Table 1:** Association between demographic variables and amniotomy exposure.

Variable	Early amniotomy	Late amniotomy	*P* value^*^
	*N* = 398	*N* = 1,169	
Maternal age, yr	23 (19–29)	22 (19–28)	0.48
African American race, *n* (%)	98 (26.3)	310 (28.3)	0.48
Chronic hypertension or preeclampsia, *n* (%)	127 (31.9)	336 (28.7)	0.23
Pregestational or gestational diabetes, *n* (%)	27 (6.8)	56 (4.8)	0.12
Obesity, *n* (%)	23 (8.3)	104 (8.9)	0.72
HIV, *n* (%)	0	0	
Lupus, *n* (%)	2 (0.5)	0	0.06
Smoking, *n* (%)	8 (2)	22 (1.9)	0.83
Location of prenatal care, *n* (%)			
Private practice	251 (63.9)	744 (65.2)	0.71
Medicaid practice	134 (34.1)	368 (32.3)	
No prenatal care	8 (2.0)	29 (2.5)	
Gestational age at delivery, wk	39.6 (39.0–41.0)	40.0 (39.1–41.0)	0.07
Neonatal weight, gms	3275 (2895–3610)	3220 (2856–3556)	0.10
Indication for induction, *n* (%)			
Maternal	27 (51.9)	227 (52.7)	0.68
Fetal	25 (48.1)	198 (48.9)	
Both	0	6 (1.4)	
Method of Induction, *n* (%)			
Pitocin	329 (82.7)	962 (82.3)	0.87
Prostaglandin	224 (56.6)	576 (49.4)	**0.01**
Foley bulb	79 (20.0)	216 (18.5)	0.53
Multiple methods	227 (57.3)	606 (52.0)	0.06
Any cervical ripening	266 (67.2)	710 (60.9)	**0.03**
Epidural anesthesia, *n* (%)	366 (94.1)	1,084 (94.1)	0.99
Maternal GBS positive, *n* (%)	135 (36.1)	377 (34.2)	0.50
Number of vaginal exams, *n* (%)	8 (6–11)	8 (6–10)	0.47
Meconium stained amniotic fluid, *n* (%)	64 (16.4)	217 (18.7)	0.30
Fetal scalp electrode, *n* (%)	181 (45.5)	395 (33.8)	**<0.001**
Intrauterine pressure catheter, *n* (%)	232 (58.3)	533 (45.7)	**<0.001**
Year of delivery, *n* (%)			
2008	133 (33.6)	230 (19.7)	**<0.001**
2009	98 (24.8)	267 (22.9)	
2010	55 (13.9)	334 (28.6)	
2011	110 (27.8)	337 (28.9)	
Cervical dilation at amniotomy (cm)	3 (3.0–3.5)	4.5 (4.0–5.2)	** <0.001**

^*^Categorical data analyzed with chi-square tests and data reported as *N* (%).

Continuous data analyzed with nonparametric comparison of medians and reported as median (interquartile range).

**Table 2:** Association between amniotomy exposure and maternal outcomes.

Outcome	Early amniotomy	Late amniotomy	*P* value^*^
	*N* = 398	*N* = 1,169	
Chorioamnionitis, *n* (%)	40 (10.2)	149 (13.2)	0.12
Rates of chorioamnionitis based on mode of delivery, *n* (%)			
Vaginal delivery	20 (8.4)	117 (14.6)	**0.01**
Cesarean section	20 (12.7)	32 (9.7)	0.30
Mode of delivery, *n* (%)			
Cesarean section	160 (40.2)	345 (29.5)	**<0.001**
Operative vaginal delivery	31 (7.8)	117 (10.0)	0.19
Time to vaginal delivery, minutes	329 (216–493)	472 (313–700)	**<0.001**
Fetal weight (gms)			
Vaginal delivery	3172 (2828–3444)	3192 (2828–3528)	0.33
Cesarean section	3332 (2856–3696)	3444 (3065–3752)	**0.02**
Endometritis, *n* (%)	9 (2.3)	11 (0.9)	**0.04**
Cord prolapse, *n* (%)	1 (0.3)	2 (0.2)	0.56
Neonatal 5-minute APGARs	9 (9-9)	9 (9-9)	0.13
Admission to intensive care nursery, *n* (%)	57 (14.3)	166 (14.2)	0.95

^*^Categorical data analyzed with chi-square tests and data reported as *N* (%).

Continuous data analyzed with nonparametric comparison of medians and reported as median (interquartile range).

**Table 3:** Association between demographic variables and chorioamnionitis.

Variable	Chorioamnionitis	No chorioamnionitis	*P* value^*^
	*N* = 189	*N* = 1,333	
Maternal age, yr	22 (19–28)	22 (19–29)	0.50
African American race, *n* (%)	52 (28.9)	341 (27.4)	0.67
Chronic hypertension or preeclampsia, *n* (%)	55 (29.1)	403 (30.2)	0.75
Pregestational or gestational diabetes, *n* (%)	6 (3.2)	76 (5.7)	0.15
Obesity, *n* (%)	11 (5.8)	121 (9.1)	0.13
HIV, *n* (%)	0	0	
Lupus, *n* (%)	0	2 (0.15)	1.0
Smoking, *n* (%)	3 (1.6)	27 (2.0)	1.0
Location of prenatal care, *n* (%)			
Private practice	110 (60.1)	857 (65.6)	**0.03**
Medicaid practice	64 (35.0)	423 (32.4)	
No prenatal care	9 (4.9)	26 (2.0)	
Gestational age at delivery, wk	40.1 (39.0–41.1)	40.0 (39.0–41.0)	0.07
Neonatal weight, gms	3248 (2856–3584)	3304 (2940–3528)	0.75
Indication for induction, *n* (%)			
Maternal	66 (34.9)	504 (37.8)	0.59
Fetal	121 (64.0)	821 (61.6)	
Both	2 (1.1)	8 (0.6)	
Method of induction, *n* (%)			
Pitocin	157 (83.1)	1,097 (82.3)	0.79
Prostaglandin	97 (51.3)	677 (51.0)	0.93
Foley bulb	39 (20.6)	247 (18.6)	0.50
Multiple methods	102 (54.0)	706 (53.2)	0.84
Any cervical ripening	115 (60.9)	832 (62.7)	0.63
Epidural anesthesia, *n* (%)	183 (97.3)	1,267 (93.6)	**0.04**
Maternal GBS positive, *n* (%)	53 (29.8)	441 (35.5)	0.14
Number of vaginal exams, *n* (%)	8 (6–10)	8 (6–11)	0.12
Meconium stained amniotic fluid, *n* (%)	43 (22.5)	238 (17.5)	0.10
Fetal scalp electrode, *n* (%)	67 (35.5)	489 (36.7)	0.74
Intrauterine pressure catheter, *n* (%)	82 (43.4)	658 (49.4)	0.12
Year of delivery, *n* (%)			
2008	50 (26.5)	306 (23.0)	0.71
2009	40 (21.2)	318 (23.9)	
2010	46 (24.3)	333 (25.0)	
2011	53 (28.4)	373 (28.0)	

^*^Categorical data analyzed with chi-square tests and data reported as *N* (%).

Continuous data analyzed with nonparametric comparison of medians and reported as median (interquartile range).

**Table 4:** Indication for cesarean section.

Indication	Early amniotomy	Late amniotomy	*P* value^*^
	*N* = 160	*N* = 345	
Labor dystocia, *n* (%)			0.8
Arrest of active phase	32 (20)	93 (27.0)	
Arrest of descent	23 (14.4)	59 (17.1)	
Failure to progress	13 (8.1)	22 (6.4)	
Failed induction of labor	12 (7.5)	12 (3.5)	
Nonreassuring fetal heart tracing (NRFHT), *n* (%)			
NRFHT/fetal intolerance of labor	74 (46.3)	126 (36.5)	
NRFHT with concurrent diagnosis of arrest	4 (2.5)	26 (7.5)	
Cord prolapse	1 (0.6)	2 (0.6)	
Other, *n* (%)			
Malpresentation	0	2 (0.6)	
Elective	1 (0.6)	2 (0.6)	
Placenta previa	0	1 (0.3)	

^*^Categorical data analyzed with chi-square tests and data reported as *N* (%).

**Table 5:** Multivariable logistic analyses. Association between early amniotomy and chorioamnionitis, controlling for potential confounders.

Variable	Odds ratio	95% CI	*P* value
Association with chorioamnionitis, among those with vaginal delivery			
Early amniotomy	0.56	0.34–0.93	**0.02**
Confounders			
Intrauterine pressure catheter	0.83	0.54–1.28	0.41
Fetal scalp electrode	0.86	0.54–1.38	0.54
Epidural anesthesia	0.94	0.69–1.27	0.68
Year of delivery	0.95	0.81–1.11	0.53
Any cervical ripening	0.90	0.62–1.30	0.58

Association with chorioamnionitis, among all patients			
Early amniotomy	0.76	0.52–1.10	0.15
Confounders			
Location of prenatal care	1.33	1.00–1.76	**0.04**
Gestational age at delivery	1.02	0.96–1.08	0.61
Epidural anesthesia	0.99	0.98–1.01	0.51
Meconium stained amniotic fluid	0.99	0.97–1.01	0.44
Any cervical ripening	0.92	0.67–1.26	0.60
